# Nitrate Increased Cucumber Tolerance to Fusarium Wilt by Regulating Fungal Toxin Production and Distribution

**DOI:** 10.3390/toxins9030100

**Published:** 2017-03-11

**Authors:** Jinyan Zhou, Min Wang, Yuming Sun, Zechen Gu, Ruirui Wang, Asanjan Saydin, Qirong Shen, Shiwei Guo

**Affiliations:** 1 Jiangsu Provincial Key Lab for Organic Solid Waste Utilization, National Engineering Research Center for Organic–based Fertilizers, Jiangsu Collaborative Innovation Center for Solid Organic Waste Resource Utilization, Nanjing Agricultural University, Nanjing 210095, China; 2011203030@njau.edu.cn (J.Z.); minwang@njau.edu.cn (M.W.); 2014203028@njau.edu.cn (Y.S.); 2014103116@njau.edu.cn (Z.G.); 2015103106@njau.edu.cn (R.W.); shenqirong@njau.edu.cn (Q.S.); 2 Center of Agricultural Technology Extension, Kizilsu Kirghiz Autonomous Prefecture 845350, China; asj.syd@163.com

**Keywords:** Fusarium wilt, cucumber, nitrogen form, nitrogen supply, fusaric acid

## Abstract

Cucumber Fusarium wilt, induced by *Fusarium oxysporum* f. sp. *cucumerinum* (FOC), causes severe losses in cucumber yield and quality. Nitrogen (N), as the most important mineral nutrient for plants, plays a critical role in plant–pathogen interactions. Hydroponic assays were conducted to investigate the effects of different N forms (NH_4_^+^ vs. NO_3_^‒^) and supply levels (low, 1 mM; high, 5 mM) on cucumber Fusarium wilt. The NO_3_^‒^-fed cucumber plants were more tolerant to Fusarium wilt compared with NH_4_^+^-fed plants, and accompanied by lower leaf temperature after FOC infection. The disease index decreased as the NO_3_^‒^ supply increased but increased with the NH_4_^+^ level supplied. Although the FOC grew better under high NO_3_^−^ in vitro, FOC colonization and fusaric acid (FA) production decreased in cucumber plants under high NO_3_^−^ supply, associated with lower leaf membrane injury. There was a positive correlation between the FA content and the FOC number or relative membrane injury. After the exogenous application of FA, less FA accumulated in the leaves under NO_3_^−^ feeding, accompanied with a lower leaf membrane injury. In conclusion, higher NO_3_^−^ supply protected cucumber plants against Fusarium wilt by suppressing FOC colonization and FA production in plants, and increasing the plant tolerance to FA.

## 1. Introduction

Cucumber Fusarium wilt, a common fungal disease caused by the soil-borne pathogen *Fusarium oxysporum* f. sp*. cucumerinum* (FOC), leads to severe losses in the production of cucumbers worldwide [1,2]. This pathogen can survive for long periods in the soil and invade cucumbers at any stage of development. The pathogen infects the roots of host plants via direct penetration or wounds and ultimately colonizes the vascular vessels by reaching the vascular tissue [3]. The visible symptoms of Fusarium wilt disease are necrotic lesions, followed by foliar yellowing, wilting, vascular tissue damage, and finally plant death [1], attributed to mycelium colonization and toxins production of the pathogen [4].

Fusaric acid (FA, 5-n-butyl-2-pyridine carboxylic acid), a well-known non-host-specific toxin produced by *Fusarium* species and isolated from diseased plant tissues, is correlated with the development of plant disease symptoms induced by the infection of some *Fusarium* species [5]. It was reported that FA is the critical causal agent of Fusarium wilt through leaf cell membrane injury [6,7]. Many studies have shown that FA disturbs the metabolism of infected plants and decreases the viability of plant cells, including the early hyperpolarization of the root membrane electrical potential [8]; alterations in membrane permeability [9]; an increase of electrolyte leakage [10,11]; the inhibition of defense enzymes and respiratory activity [12,13]; and a decrease in ATP levels and stomatal conductance [14,15], thus inhibiting plant growth and eventually leading to death [15,16,17]. Generally, the disease index was positively correlated with FA production [18].

Nitrogen (N), a key element for plant metabolism, has long been demonstrated to influence plant-microbe interactions and plant disease development [19,20,21,22]. It is well known that different N forms can affect the physiological process of plants, such as enzyme activity [23], photosynthetic rate [24], respiration rate [25], water balance [26], and the signaling pathway [27], thus eventually influencing plant diseases. Moreover, different N forms could also regulate disease tolerance by affecting pathogen metabolic adaptation and signals controlling virulence factor activation [28,29]. For instance, ammonium represses *Fusarium* infection by controlling virulence [29]. Additionally, N supply levels are also related to host resistance, and several studies have found that a high N supply could decrease plant resistance to diseases [30] by increasing nutrients for pathogen development [22,31,32]. However, other reports have suggested a decreased resistance of plants to diseases with a lower N supply [33] resulted from a higher C/N and lower nitrogenous compounds (e.g., protein), which are involved in plant resistance to disease [34]. In addition, N limitation has been presented as a key signal for triggering the expression of virulence genes in plant pathogens, thus influencing plant diseases [35]. Overall, the diverse effects of N on disease development are inconsistent and depend on the host species, pathogen type, N forms and supply, and the timing of the N application [19,36,37].

Although the individual effects of different N forms or N supply levels on plant disease development have been widely studied, the combined effects of N forms and supply levels on plant–pathogen interactions are largely unknown. Moreover, several studies have reported that the toxin produced by fungi in vitro is regulated by N [38], while the effects of N on the toxin production and accumulation of infected plants are not well understood. In our present study, hydroponic experiments were conducted to identify the influence of N forms and supply levels on cucumber tolerance to Fusarium wilt. We characterized the influence of N forms and supply levels on disease index dynamic changes, cucumber growth and leaf temperature changes to identify the parameters of the interaction that are affected by N supply. Moreover, our previous studies reported that FA plays a critical role in Fusarium wilt by causing leaf cell membrane injury [6,7]. Thus, FA was applied in the nutrient solution to further investigate the role of FA on Fusarium wilt in response to different N sources. We mainly focused on the relationships among the N content, FOC number, FA accumulation, and leaf relative membrane injury to seek the preliminary cause.

## 2. Results

### 2.1. Effects of Different N Sources on the Growth and Disease Index of Cucumber Plants

**Figure 1:**
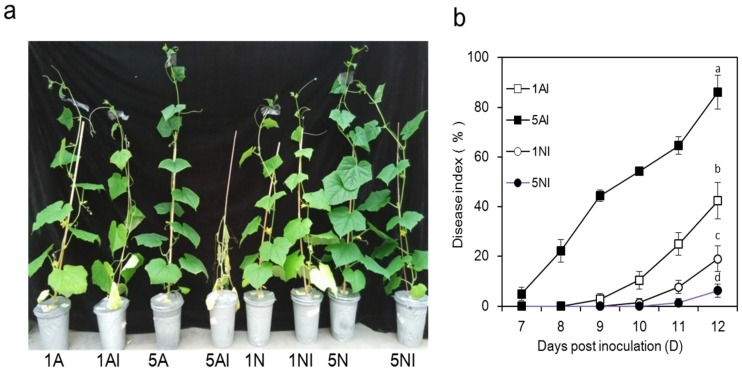
The disease symptoms and disease index of Fusarium wilt of cucumber plants caused by *Fusarium oxysporum* f. sp*. cucumerinum* with different nitrogen sources. 1A, 5A (non-inoculated control plants with 1 mM or 5 mM NH_4_^+^, respectively); 1N, 5N (non-inoculated control plants with 1 mM or 5 mM NO_3_^−^, respectively); 1AI, 5AI (inoculated plants with 1 mM or 5 mM NH_4_^+^, respectively); and 1NI, 5NI (inoculated plants with 1 mM or 5 mM NO_3_^−^, respectively). (**a**) Disease symptoms at 12 days post inoculation; and (**b**) development of the disease index of Fusarium wilt of cucumber plants. The disease index was calculated from 7 to 12 days post inoculation. Error bars indicate the standard deviations from 20 plants for each treatment. Experiments were repeated more than three times with similar results. The data are the means ± SD of four replicates. Significant differences (LSD test, *p* < 0.05) at 12 days post inoculation among treatments are indicated by different letters.

Cucumber plants infected by *Fusarium oxysporum* f. sp*. cucumerinum* (FOC) differed notably in their disease symptoms at 12 days post inoculation when the plants were at the flowering stage (Figure 1a). Compared with NO_3_^−^-fed plants, the disease symptoms of NH_4_^+^-fed plants were more severe, corresponding to obvious yellowing and wilt. As the N supply increased, the disease index of NO_3_^−^-fed plants significantly decreased, while that of NH_4_^+^-fed plants markedly increased (Figure 1b). In fact, the disease index gradually increased with the extension of the cultivation period in all the FOC-inoculated-treatments, and at 12 days post inoculation, the levels were 42.6%, 86.1%, 18.8% and 6.5% in the 1AI, 5AI, 1NI and 5NI treatments, respectively (Figure 1b).

As shown in Table 1, under non-inoculated conditions, the dry weights of the roots, stems and leaves of high-NO_3_^−^-fed cucumber plants increased by approximately 17%, 36%, and 48%, respectively, compared with high-NH_4_^+^-fed plants (Table 1). However, those of the low-NO_3_^−^-fed plants increased by approximately 11%, 16%, and 17%, respectively, compared with low-NH_4_^+^-fed plants, and the dry weights increased with the N supply increase (Table 1). After FOC inoculation, the dry weights of the roots, stems and leaves of high-NH_4_^+^-fed plants were significantly reduced by approximately 51%, 29%, and 30%, respectively, while the low-NH_4_^+^-fed plants were reduced by approximately 13%, 11% and 30%, respectively. In the NO_3_^−^ treatments, these parameters were not significantly affected.

### 2.2. Effects of Different N Sources on the Leaf Temperature of Cucumber Plants

**Figure 2:**
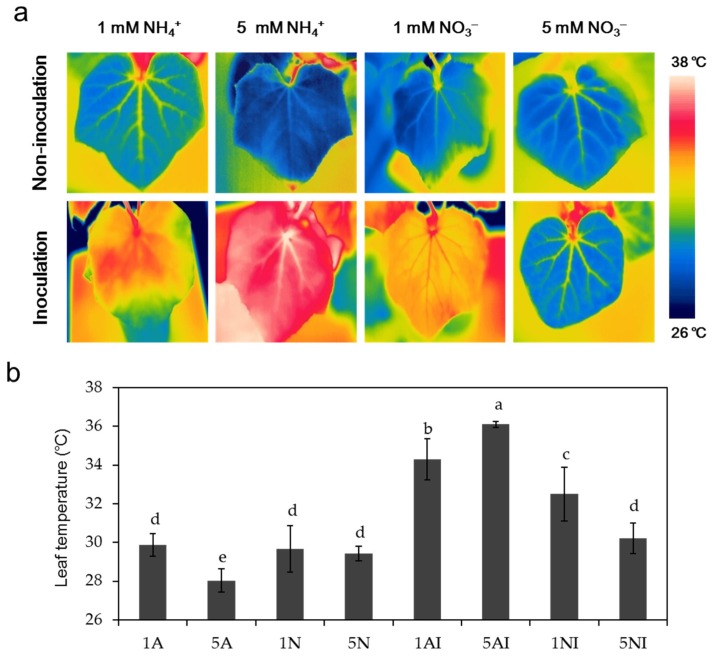
Effects of different nitrogen sources and FOC inoculation on the leaf temperature of cucumber plants: (**a**) thermal images of cucumber plants at 12 days post inoculation in the light; and (**b**) leaf temperatures of cucumber plants after FOC inoculation. 1A, 5A (non-inoculated control plants with 1 mM or 5 mM NH_4_^+^, respectively); 1N, 5N (non-inoculated plants with 1 mM or 5 mM NO_3_^−^, respectively); 1AI, 5AI (inoculated plants with 1 mM or 5 mM NH_4_^+^, respectively); and 1NI, 5NI (inoculated plants with 1 mM or 5 mM NO_3_^−^, respectively). Thermographics were performed on the new fully expanded leaf per plant using four replicates per treatment. The data are the means ± SD of four replicates. Significant differences (LSD test, *p* < 0.05) among treatments are indicated by different letters.

The average leaf temperature was measured in light at 12 days post inoculation (Figure 2a,b). Under non-inoculation conditions, the leaf temperature in high-NH_4_^+^-fed cucumber plants was lower than that in low-NH_4_^+^-fed plants, and no significant difference was found between plants treated with different NO_3_^−^ supplies. After FOC inoculation, the average leaf temperatures of plants supplied with low NH_4_^+^ and high NH_4_^+^ increased by 15% and 29%, respectively, compared with the non-inoculation treatment. The temperature of low-NO_3_^−^-fed plants increased by approximately 10%, while no significant change was observed between plants with a high NO_3_^−^ supply.

### 2.3. Effect of Different N Sources on the Pathogen Growth In Vitro Culture

**Figure 3:**
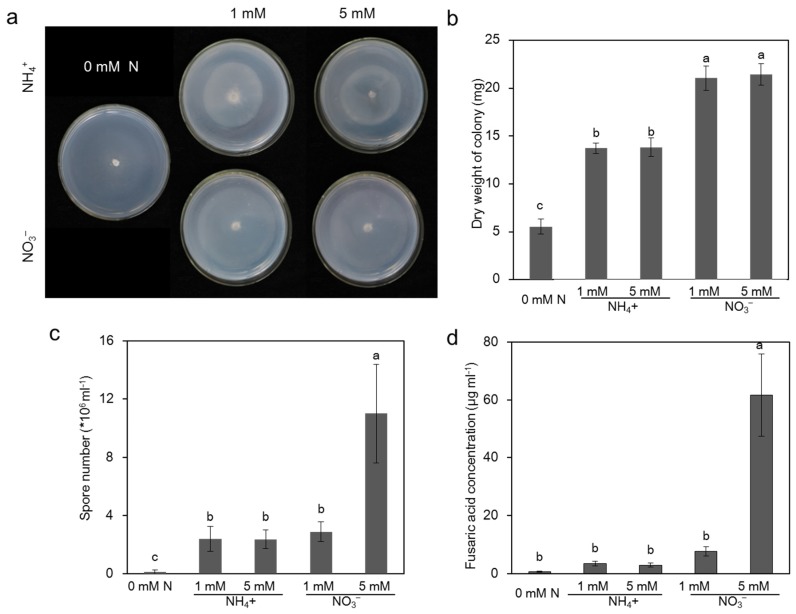
Effects of different nitrogen sources on pathogen growth in vitro culture. (**a**,**b**) The colony growth of the *Fusarium oxysporum* f. sp. *cucumerinum* grown on medium without N (0 mM N) and with NH_4_^+^ (1 mM or 5 mM) or NO_3_^−^ (1 mM or 5 mM). Pictures were taken on the seventh day of inoculation. (**c**) The spore number of the pathogen grown on medium without N (0 mM N) and with NH_4_^+^ (1 mM or 5 mM) or NO_3_^−^ (1 mM or 5 mM). The conidia were counted using a hemocytometer on the seventh day of inoculation. (**d**) The extracted FA produced by the pathogen on medium without N (0 mM N) and with NH_4_^+^ (1 mM or 5 mM) or NO_3_^−^ (1 mM or 5 mM). The NH_4_^+^ was added in the form of (NH_4_)_2_SO_4_, and NO_3_^−^ was added as Ca(NO_3_)_2_. All of the experiments were repeated more than three times with similar results. The data are the means ± SD of four replicates. Significant differences (LSD test, *p* < 0.05) among treatments are indicated by different letters.

To investigate the effects of rhizosphere environment on the pathogen propagation and infection, we investigated the effects of different N sources in the hydroponic nutrition solution on pathogen growth in vitro. Interestingly, seven days after incubation, compared with the medium supplied with NH_4_^+^, the colony diameter in the medium without N was significantly increased, but the colony density was significantly sparse (Figure 3a). Therefore, we measured the colony dry weight to reflect the pathogen growth. The pathogen colony dry weight under no N supply in the medium was lower than that of the medium supplied with N, and there was no significant difference under different N concentrations (1 mM vs. 5 mM) regardless of the N forms (Figure 3a,b). When supplied with the same N concentration, NO_3_^−^ increased the colony dry weight by approximately 55% compared with the NH_4_^+^ treatment (Figure 3a,b). The spore number and toxin of the pathogen were correlated with the disease severity [3,6]. Subsequently, we assessed the spore number and extracted FA on a medium with a different N source. As shown in Figure 3c, the pathogen spore number of medium without N was lower than that of medium with N, and it was increased by approximately 279% in the medium with high NO_3_^−^ compared with that of low NO_3_^−^, while no significant difference was found between the media with different NH_4_^+^ sources (Figure 3c). The extracted FA content of the medium with high NO_3_^−^ was significantly higher than the medium without N. In addition, the extracted FA content of the medium with a high NO_3_^−^ source was almost eight-fold higher than that of the medium with low NO_3_^−^ (Figure 3d), and no significant difference was found between the media with different NH_4_^+^ sources (Figure 3d).

### 2.4. Effects of Different N Sources on the NH_4_^+^ and NO_3_^−^ Content of Cucumber Plants

**Figure 4:**
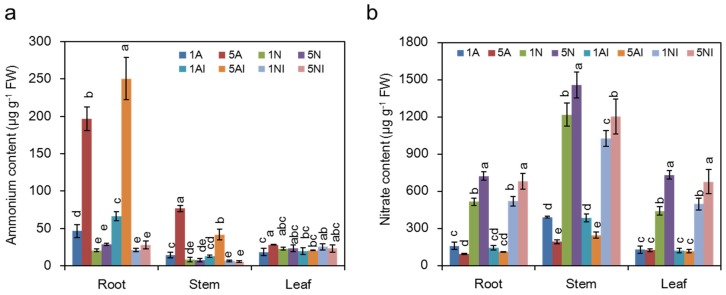
Effects of different nitrogen sources on the ammonium (**a**) or nitrate content (**b**) in cucumber plants after FOC inoculation. 1A, 5A (non-inoculated control plants with 1 mM or 5 mM NH_4_^+^, respectively); 1N, 5N (non-inoculated plants with 1 mM or 5 mM NO_3_^−^, respectively); 1AI, 5AI (inoculated plants with 1 mM or 5 mM NH_4_^+^, respectively); and 1NI, 5NI (inoculated plants with 1 mM or 5 mM NO_3_^−^, respectively). The data are the means ± SD of four replicates (*p* < 0.05). Significant differences (LSD test, *p* < 0.05) of the same parts of the plants among treatments are indicated by different letters.

As shown in Figure 4a, under non-inoculation conditions, the ammonium content in high-NH_4_^+^-fed plants was significantly higher compared to low NH_4_^+^-fed plants. After FOC inoculation, the NH_4_^+^ content under NH_4_^+^ nutrition significantly increased in the roots, but no significant difference was found in plants with different NO_3_^−^ supplies.

Under non-inoculation conditions, the NO_3_^−^ content in high-NO_3_^−^-fed plants was significantly higher than that in low-NO_3_^−^-fed plants (Figure 4b). After FOC inoculation, the NO_3_^−^ content under NO_3_^−^ nutrition decreased in the stems, and there was no significant difference in the NH_4_^+^-fed plants after FOC inoculation.

### 2.5. Effect of Different N Sources on the Pathogen Distributions and FA Content of Cucumber Plants

**Figure 5:**
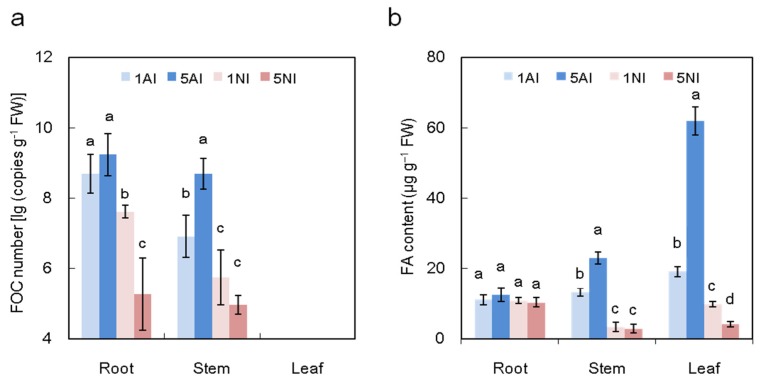
Effects of different nitrogen sources on the FOC distribution (**a**) and FA content (**b**) in cucumber plants after FOC inoculation. 1AI, 5AI (inoculated plants with 1 mM or 5 mM NH_4_^+^, respectively); and 1NI, 5NI (inoculated plants with 1 mM or 5 mM NO_3_^−^, respectively). FOC could not be detected in the non-inoculated plants. The data are the means ± SD of four replicates. Significant differences (LSD test, *p* < 0.05) of the same parts of the plants among treatments are indicated by different letters.

As shown in Figure 5a, 12 days post inoculation, FOC could be detected in the roots and stems but not in leaves of plants of any of the treatments. Moreover, the number of FOC in the roots and stems was lower in the NO_3_^−^ treatments than in the NH_4_^+^ treatments, regardless of the N concentration, and it was lowest in the high-NO_3_^−^-fed plants. When compared with low-NH_4_^+^-fed plants, the FOC number in high-NH_4_^+^-fed plants increased by 26% in the stems. However, with the NO_3_^−^ supply increase, the FOC number of the plants decreased by approximately 31% in the roots.

We measured the FA production at 10 days post inoculation when the wilt symptoms occurred in low-NO_3_^−^-fed plants. FA production could not be detected in non-inoculated control plants. As shown in Figure 5b, after inoculation, the FA content was much lower in NO_3_^−^-fed plants than in NH_4_^+^-fed plants. With the increased N supply, the FA content of NH_4_^+^-fed plants increased by approximately 74% and 224% in the stems and leaves, respectively, while that of the NO_3_^−^-fed plants decreased by approximately 58% in the leaves.

### 2.6. Correlations between the NH_4_^+^ Content or NO_3_^−^ Content and the FOC Number and between the Total FOC Number and the Total FA Content or Disease Index

**Figure 6:**
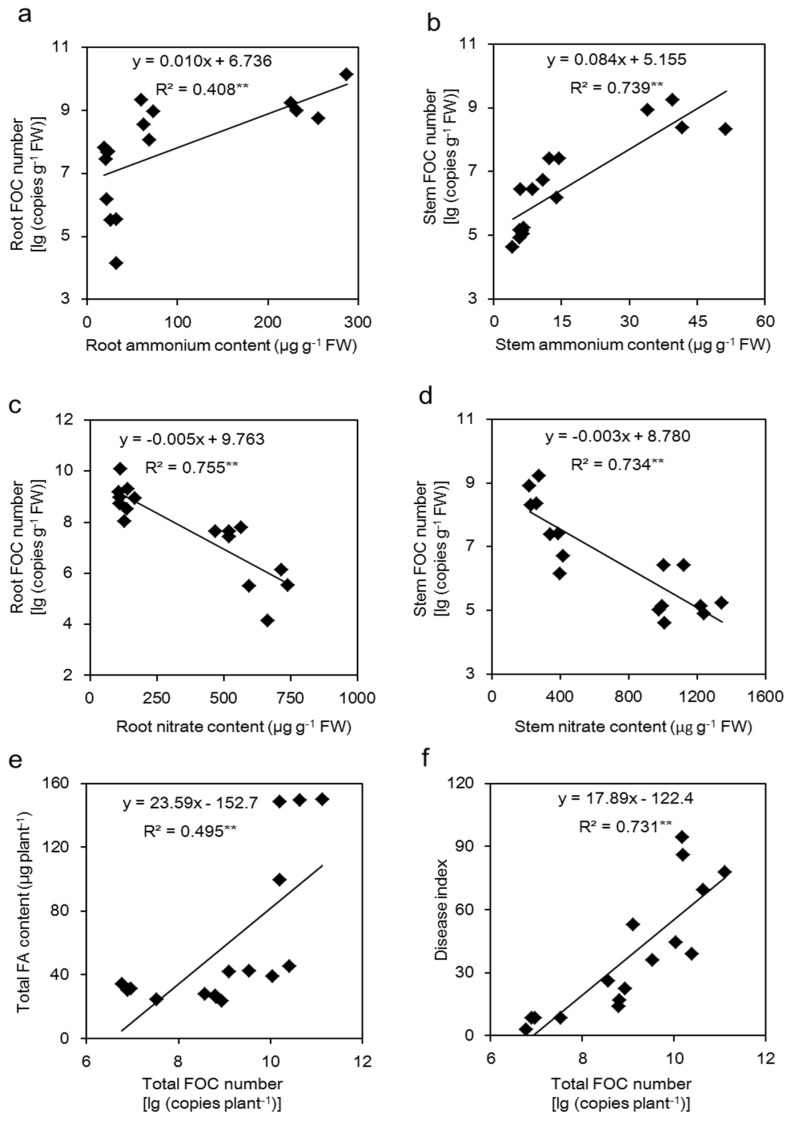
Correlations between the ammonium content (**a**,**b**) or nitrate content (**c**,**d**) and the FOC number of the roots and stems, and between the total FOC number and the total FA content of the plants (**e**) or disease index (**f**). The regression lines are shown in the figures.

In the roots and stems, the NH_4_^+^ content was positively correlated with the FOC number (*p* < 0.01) (Figure 6a,b), while the NO_3_^−^ content was negatively related to the corresponding FOC number (*p* < 0.01) (Figure 6c,d). A significant positive correlation (*p* < 0.01) was found between the total FOC number and the total FA content (Figure 6e), and we also found that the total FOC number was positively correlated with the disease index (Figure 6f).

### 2.7. Effect of Different N Sources on the Leaf Relative Membrane Injury of Cucumber Plants after FOC Infection

**Figure 7:**
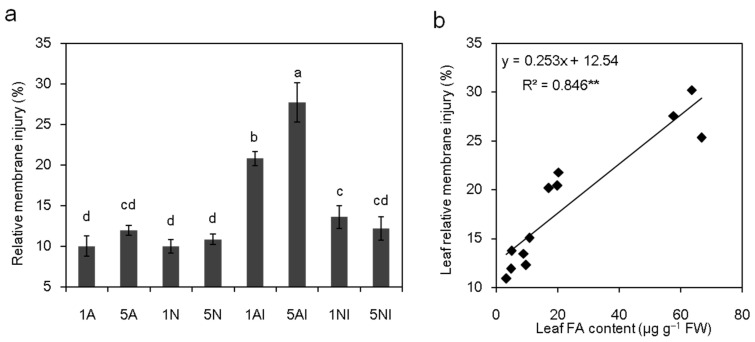
Analysis of the relationship between leaf relative membrane injury and FA content in cucumber plants: (**a**) effects of different nitrogen sources on the relative membrane injury of cucumber plants after FOC inoculation; and (**b**) correlation between the leaf FA content and leaf relative membrane injury of cucumber plants. 1A, 5A (non-inoculated control plants with 1 mM or 5 mM NH_4_^+^, respectively); 1N, 5N (non-inoculated control plants with 1 mM or 5 mM NO_3_^−^, respectively); 1AI, 5AI (inoculated plants with 1 mM or 5 mM NH_4_^+^, respectively); and 1NI, 5NI (inoculated plants with 1 mM or 5 mM NO_3_^−^, respectively). The regression line is shown in the figure. The data are the means ± SD of four replicates. Significant differences (LSD test, *p* < 0.05) among treatments are indicated by different letters.

Under non-inoculation conditions, there was no significant difference in the leaf relative membrane injury among all the treatments (Figure 7a). After FOC inoculation, the leaf relative membrane injury of the low-NH_4_^+^-fed and high-NH_4_^+^-fed plants increased by 107% and 132%, respectively, whereas that of low-NO_3_^−^-fed plants increased by 36%; no significant difference was found in high-NO_3_^−^ plants (Figure 7a). A significant positive correlation (*p* < 0.01) was observed between the leaf FA content and the leaf relative membrane injury (Figure 7b).

### 2.8. Effect of FA on the Cucumber Plants Treated with Different N Sources

**Figure 8:**
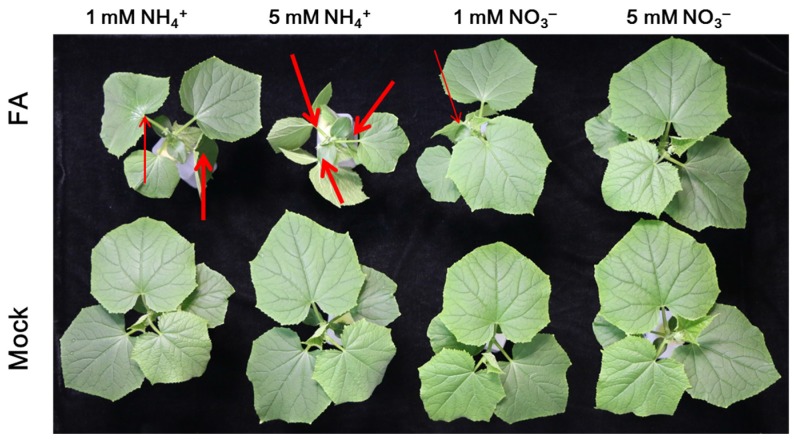
Effects of FA on cucumber plants treated with different nitrogen sources. Plants were treated with 60 ppm FA or were mock treated. Photos were taken 9 h after FA treatment. The arrow pointed to the wilting leaves, and the wilting degree was increased with the increased thickness of arrow. Experiments were repeated more than three times with similar results.

The effect of FA application on cucumber plants treated with different N sources is shown in Figure 8, and it is consistent with those observed regarding the effects of FOC infection (Figure 1). No wilt symptoms were observed in the leaves of the mock-treated plants. After FA treatment, as the N supply increased, the wilt symptoms of the plants supplied with NO_3_^−^ were markedly alleviated, while those of the NH_4_^+^-fed plants were obviously aggravated (Figure 8).

**Figure 9:**
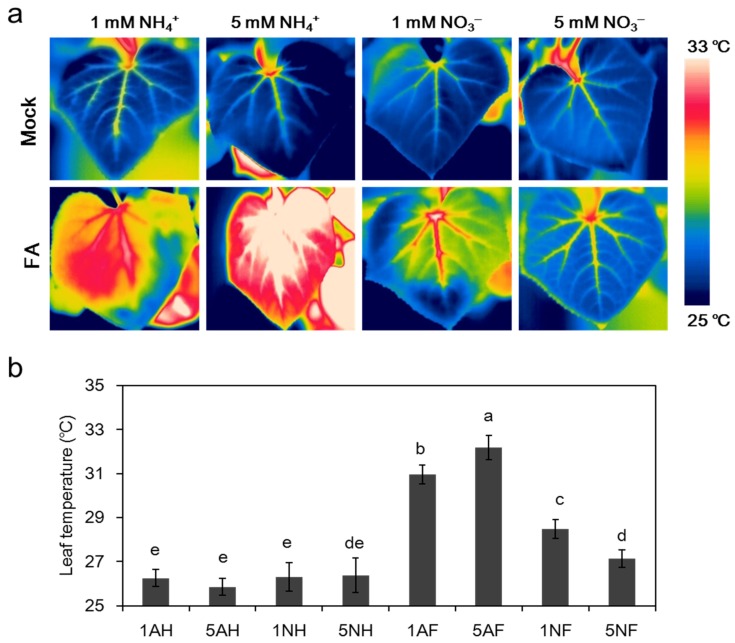
Effects of FA on the leaf temperature of cucumber plants treated with different nitrogen sources: (**a**) thermal images were taken in the light at 7 h after FA treatment when wilt symptoms occurred in 1 mM NO_3_^−^-fed plants; and (**b**) leaf temperature in cucumber plants after FA treatment. 1AH, 5AH (mock-treated 1 mM or 5 mM NH_4_^+^-fed plants, respectively); 1NH, 5NH (mock-treated 1 mM or 5 mM NO_3_^−^-fed plants, respectively); 1AF, 5AF (60 ppm FA-treated 1 mM or 5 mM NH_4_^+^-fed plants, respectively); 1NF, 5NF (60 ppm FA-treated 1 mM or 5 mM NO_3_^−^-fed plants, respectively). The thermographics were performed on the new fully expanded leaf per plant using four replicates per treatment. The data are the means ± SD of four replicates. Significant differences (LSD test, *p* < 0.05) among treatments are indicated by different letters.

As shown in Figure 9, without FA treatment, there was no significant difference between any of the treatments. After FA treatment, the average leaf temperature of the plants supplied with low NH_4_^+^ and high NH_4_^+^ increased by 18% and 24%, respectively, while that of the low-NO_3_^−^-fed plants increased slightly by 8%. No significant difference was observed under high NO_3_^−^ supply.

**Figure 10:**
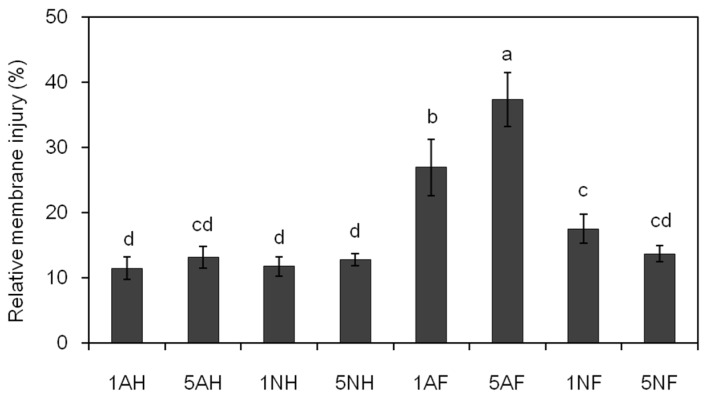
Effects of FA on the relative membrane injury of cucumber plants treated with different nitrogen sources. The membrane injury of the leaves was measured at 7 h after FA treatment when wilt symptoms occurred in 1 mM NO_3_^−^-fed plants. 1AH, 5AH (mock-treated 1 mM or 5 mM NH_4_^+^-fed plants, respectively); 1NH, 5NH (mock-treated 1 mM or 5 mM NO_3_^−^-fed plants, respectively); 1AF, 5AF (60 ppm FA-treated 1 mM or 5 mM NH_4_^+^-fed plants, respectively); 1NF, 5NF (60 ppm FA-treated 1 mM or 5 mM NO_3_^−^-fed plants, respectively). The data are the means ± SD of four replicates. Significant differences (LSD test, *p* < 0.05) among treatments are indicated by different letters.

Without FA treatment, no significant difference in the leaf relative membrane injury of the cucumber plants was observed with the different N sources (Figure 10). After FA treatment, the leaf relative membrane injury of NH_4_^+^-fed plants increased by 134% and 183%, respectively, with the increased N supply, while that of low-NO_3_^−^-fed plants increased by approximately 49%, and no significant difference was found in high-NO_3_^−^-fed plants (Figure 10).

The FA distribution was detected in the roots, stems and leaves of FA-treated cucumber plants when wilt symptoms occurred in 1 mM NO_3_^−^-fed plants (Table 2). Compared to NH_4_^+^-fed plants, the FA in NO_3_^−^-fed plants mainly accumulated in the roots after FA infection. The FA content of high-NH_4_^+^-fed plants increased by 23% in the leaves compared with low-NH_4_^+^-fed plants, while the FA content of high-NO_3_^−^-fed plants increased by 13% in the roots and decreased by 17% in the leaves compared to that of the low-NO_3_^−^-fed plants.

## 3. Discussion

Nitrogen (N), which is necessary for the normal growth of plants, greatly influences disease development and plant resistance. Documentations have shown that nitrate nitrogen (NO_3_^−^) decreased the root rot caused by *Fusarium oxysporum* in bean and wheat and the crown rot of sugar beets induced by *Rhizoctonia* *solani*. However, ammonium nitrogen (NH_4_^+^) suppresses the take–all of wheat induced by *Ophiobolus* and the root rot of corn caused by *Pythium* [20,39]. In the present study, NO_3_^−^ nutrition increased the resistance of cucumber plants to Fusarium wilt compared with NH_4_^+^ nutrition (Figure 1a,b). It has been reported that plant resistance to diseases could be regulated by N forms [20,22], and different N sources could change the physiological and biochemical processes of the plant, thus affecting plant–pathogen interactions [19]. For example, Fernández-Crespo et al. [40] observed that NH_4_^+^ decreased the susceptibility of tomatoes to *Pseudomonas syringae* because of the activation of systemic acquired acclimation. In contrast, compared to the NH_4_^+^, NO_3_^−^ increased tobacco resistance to *Pseudomonas syringae* pv. *phaseolicola* by the accumulation of SA and NO, as well as the increase in polyamine-mediated HR-linked defence [41]. In addition, studies have reported that a high N supply generally increases the susceptibility of plants to obligate parasites but decreases the susceptibility of plants to necrotrophs and facultative parasites [19,35]. Our results showed that the disease index of cucumber plants decreased with the increased NO_3_^−^ supply, but increased with the increased NH_4_^+^ supply (Figure 1b). After FOC infection, the plant biomass of NO_3_^−^-fed plants was higher than NH_4_^+^-fed plants, corresponding with a lower disease index (Table 1, Figure 1b). Lemmens et al. [42] indicated that an increased N supply could cause an increased incidence of wheat scab, which was induced by the increasing level of mycotoxin produced by *Fusarium culmorum*. However, Dietrich et al. [34] reported that the constitutive levels and the induced level of chitinase and peroxidase activity involved in the disease resistance of *Arabidopsis thaliana* were significantly higher under high N supply. Moreover, it was reported that high N levels decreased the susceptibility of tomatoes to *B. cinerea* because of the lower C/N and soluble carbohydrates [36]. Altogether, the effects of N forms and supply levels on plant diseases have been inconsistent [19,28], and depend on the host species, pathogenic microbes type and environments [22].

Leaf temperature, which could be detected by infrared imaging, changes after pathogen inoculation and can be used to monitor the development of plant diseases [43]. Several studies have reported that leaf temperature is negatively correlated with the transpiration rate [43,44]. In our study, under non-inoculated conditions, the leaf temperature of high-NH_4_^+^-fed plants was lower than NO_3_^−^-fed plants (Figure 2), which was probably caused by the higher transpiration rate [45]. After inoculation, the temperature was higher in NH_4_^+^-fed plants with a higher disease index (Figure 1b and Figure 2). This is similar to the results of Oerke et al. [43], who reported that the leaf temperature was positively correlated with the severity of the Fusarium wilt within a certain range of damage. The different leaf temperature after inoculation may due to the different abscisic acid (ABA) content in response to the different N forms and supplies [46]. Generally, pathogen infection can induce stomatal closure [47,48], which is mediated by ABA content [7,49], thus decreasing the transpiration rate and increasing the leaf temperature [50,51].

Plant–pathogen interactions are complex. When pathogens enter the host plants, they encounter new and changing nutritional environments that cause them to adopt new trophic behavior [51]. Mineral elements reduce disease incidence mostly by indirectly improving the nutritional status of host plants, which would enhance their ability to resist pathogens, or by directly inhibiting pathogen growth and activity [19]. In this work, to investigate the effects of the rhizosphere environment on the pathogen propagation and infection, the effects of different N forms and supplies of the hydroponic medium on FOC were studied in vitro. The results illustrated that FOC grew better with the NO_3_^−^ supply than the NH_4_^+^ supply (Figure 3), which is consistent with a previous study by López-Berges et al. [29], which found ammonium repressed *Fusarium oxysporum* infection-related functions (vegetative hyphal fusion and cellophane penetration) via the protein kinase TOR and the bZIP protein MeaB. Although FOC grew better under high NO_3_^−^ in vitro (Figure 3), the disease index of Fusarium wilt decreased in vivo in cucumber plants under high NO_3_^−^ supply (Figure 1), suggesting that the effects of N forms on disease development were different under in vitro and in vivo conditions. In vitro, N is a nutrient component or signaling molecule that affects pathogen growth and virulence-related function, but in vivo, N not only influences pathogen growth and virulence but also affects plant growth and metabolism, thus influencing plant disease [19]. Therefore, we mainly focused on the pathogen number in the plants and the toxin produced by the pathogen in vivo.

When plants are infected by pathogens, the ability to prevent the colonization and reproduction of the pathogen is important for their resistance. In this study, the pathogen number was significantly higher in the roots and stems of NH_4_^+^-fed plants compared with NO_3_^−^-fed plants (Figure 5a). Additionally, the FOC number was positively related to the NH_4_^+^ content but negatively related to the NO_3_^−^ content (Figure 6a–d). The N available for pathogen growth comes from plant sources, such as NH_4_^+^, NO_3_^−^ and amino acids [52]. It was reported that a large number of amino acids, such as glutamine, glutamate, alanine and γ-aminobutyric acid (GABA), can be sufficient to support pathogen growth [28,53]. Amino acids in plants could be regulated by different nitrogen forms; generally, the levels of amino acids in NH_4_^+^-fed plants are higher than NO_3_^−^-fed plants [54], thus increasing FOC colonization in high NH_4_^+^-fed plants (Figure 5a).

Fusaric acid (FA), the main fungal toxin produced by FOC, plays a vital role in Fusarium wilt development [15,49]. Although no pathogen was observed in the leaves of infected plants, FA was detected in cucumber leaves with wilt (Figure 5a,b), indicating that FA can be transported and distributed throughout the entire plant after pathogen inoculation. Fungal toxin production could be regulated by different nitrogen sources. In our results, higher NO_3_^−^ supply suppressed FA production, whereas higher NH_4_^+^ induced FA production (Figure 5b), which may because: (i) The FOC number of NO_3_^−^-fed plants was lower than NH_4_^+^-fed plants (Figure 5a), and there was a positive correlation between the FOC number and accumulated FA (Figure 6e,f). (ii) The perception of pathogens to the N nutritional status can be administer to activate the signals controlling of virulence factors and adapt the metabolic process [35,38]. Bolton and Thomma [38] reported that the virulent gene expression of *C. fulvum* can be activated by a global N regulator (transcription factor AreA/Nit2, which belongs to the GATA family of transcription factors). (iii) The amino acid metabolites regulated by N might support fungi for toxin production. However, plant N metabolism under different N forms and supplies is complicated; the cause of different FA content in the treatments needs to be further studied.

Several lines of evidence indicate that pathogen infection injures the membrane system by decreasing the activity of the plant plasma membrane H^+^-ATPase [49,55,56]. A similar result was also obtained in the present study that leaf membrane injury, which was positively correlated with the FA content (Figure 7b), remarkably increased after FOC inoculation (Figure 7a), suggesting that the wilt of cucumber plants after FOC infection was induced by FA. As the crude FA isolated from the pathogen and pure FA had the similar effects as those with the pathogen infection in plant wilts [6,57,58], we carried out experiments with the application of pure FA to further seek how it influences the Fusarium wilt of plants treated with different N sources. Our results clearly showed that the development of wilt symptoms and the change in leaf temperature were similar to those under pathogen inoculation (Figure 8 and Figure 9). In addition, leaf membrane injury after FA addition was significantly increased compared to that of mock-treated plants, and the extent of increased relative membrane injury increased with the severity of wilt (Figure 8 and Figure 10), which was similar to pathogen infection (Figure 1 and Figure 7a). Most studies have indicated that FA disordered the membrane systems of plants [10,11,15]. For instance, D’Alton and Etherton [15] reported that FA could alter the membrane potential of tomato root hairs. In addition, FA was also observed to induce the early hyperpolarization of the root membrane electrical potential [9].

To investigate the tolerance mechanisms of cucumber plants to FA, the FA distribution of the plants treated with different N sources were measured. The results showed that FA mainly accumulated in the roots of the NO_3_^−^-fed plants and was increased with a NO_3_^−^ supply (Table 2), which might contribute to the increased tolerance of NO_3_^−^-fed plants to the toxin, thus limiting FA transport to the ground and reducing damage to the leaf. Moreover, the speculation that the high-NO_3_^−^-fed plants were more tolerant to FA might be attributed to the increase in the level of defense-associated NO, a signaling molecule that is involved in the kinetics of hypersensitive response (HR) formation [59] and aids the initiation of the SA-dependent gene expression [60]. Additionally, the high-NH_4_^+^-fed plants that were more susceptible to FA may result from decreased polyamine levels, which are known to increase plant resistance by producing hydrogen peroxide (H_2_O_2_) [52].

In summary, our study revealed that higher NO_3_^−^ nutrition increased cucumber resistance to Fusarium wilt by suppressing FOC colonization and FA production in plants and increasing plant tolerance to FA. The FA may be responsible for disease development under different N sources, but the underlying mechanisms are still largely unknown. Accordingly, the interactions of FOC infection and FA production regulated by different N sources, as well as the related resistance mechanisms (e.g., amino acid, carbohydrates, hormones, proteins, NO, polyamine, etc.) should be further investigated in future studies.

## 4. Materials and Methods

### 4.1. Plant Material and Growth Conditions

Seeds of cucumber (cultivar “Jinyan 4”, susceptible to Fusarium wilt [49]) obtained from the Vegetable Research Institute of Tianjin, China, were germinated in steam-sterile quartz sand and then transplanted into plastic pots containing 1 L of aerated pre-culture nutrient solution when the first leaves emerged. The pre-culture treatment was applied in the following order: 1/8-full concentration pre-culture nutrient solution for 4 days, then 1/4-full concentration pre-culture solution for another 8 days. The full pre-culture nutrient solution was composed of 2.5 mM (NH_4_)_2_SO_4_, 2.5 mM Ca(NO_3_)_2_·4H_2_O, 1.0 mM KH_2_PO_4_, 2.0 mM K_2_SO_4_ 2.0 mM MgSO_4_·7H_2_O, 46 μM H_3_BO_3_, 11.4 μM MnCl_2_·4H_2_O, 0.7 μM ZnSO_4_·7H_2_O, 0.3 μM CuSO_4_·5H_2_O, 0.38 μM H_2_MoO_4_·4H_2_O, and 35.8 μM EDTA-Fe. A nitrification inhibitor (dicyandiamide) was added to each nutrient solution to prevent oxidation of NH_4_^+^. After pre-culture treatments, the nutrient solution was replaced by a 1/2-full concentration pre-culture nutrient solution withdraw N, and N was applied alone as either (NH_4_)_2_SO_4_ or Ca(NO_3_)_2_ for the treatment. In our previous study, we chose three concentrations of N (0.5 mM, 4 mM and 6 mM) to determine the effects of different N forms and concentrations on cucumber Fusarium wilt. We found that the concentration of 6 mM NH_4_^+^ was too high and had slight NH_4_^+^ toxicity on cucumber plants, and the concentration of 0.5 mM N was too low, as the cucumber plants could not grow normally. Therefore, we used the concentrations of 1 mM and 5 mM as the experimental condition in the present study. The treatments were as follows: 1A, 5A (non-inoculated control plants with 1 mM or 5 mM NH_4_^+^, respectively); 1N, 5N (non-inoculated plants with 1 mM or 5 mM NO_3_^−^, respectively); 1AI, 5AI (inoculated plants with 1 mM or 5 mM NH_4_^+^, respectively); and 1NI, 5NI (inoculated plants with 1 mM or 5 mM NO_3_^−^, respectively). The nutrient solutions were completely renewed every 4 days and pH was kept between 6.8 and 7.2 by the addition of CaCO_3_. The plants were cultured in a green house at 30 °C/25 °C (day/night) with a 14 h light period (photosynthetic photon flux density > 300 μmol·m^−2^·s^−1^) and a relative of humidity (RH) of 70% ±10%.

The cucumber plants examined in this study did not exhibit any visible symptoms of NH_4_^+^ toxicity, such as marginal necrosis and interveinal chlorosis on the leaves, wilting, stunted root growth or brownish roots.

### 4.2. Thermal Imaging

Infrared imaging was performed according to the methods of Wang et al. [61]. New fully expanded leaves of each plant of the same developmental stage were measured. Infrared images were obtained using an infrared camera (SC620, FLIR Systems, Inc., Wilsonville, OR, USA) with a spectral sensitivity ranging from 7.5 to 13 μm and a spatial resolution of 0.65 mrad (640 × 480 pixels focal plane array and 24° × 18° field-of-view lens with a minimum focal distance of approximately 0.3 m). The thermal resolution of the camera was 0.065 °C at 30 °C ambient temperature. Digital thermograms were analysed using the Therma CAM Researcher Professional 2.9 software (FLIR Systems, Inc., Wilsonville, OR, USA).

### 4.3. Plant Biomass Measurements

Twelve days after treatments, cucumber plants were harvested and separated into root, stem and leaf fractions. All of the samples were oven-dried at 105 °C first for 30 min and then 70 °C to constant weight. After cooling to room temperature, the dry weight of the sample was determined.

### 4.4. Fungal Colony Dry Weight, Conidial Number and Pathogen Crude Toxin Levels Grown under Different N Sources

To determine the effect of different N forms and supplies of the hydroponic nutrient solution on the pathogen growth in vitro, we used different nutrient solution that were fed to plants as the media, which contained NH_4_^+^ or NO_3_^−^ at 1 mM or 5 mM, respectively, NH_4_^+^ was added in the form of (NH_4_)_2_SO_4_, and NO_3_^−^ was added as Ca(NO_3_)_2_, and the nutrient solution without N was used as a control. The pH value of the media was adjusted to 6.8 before autoclaving, and disposable 9.0-cm-petri dishes containing 20 mL of media were used. Then, 8-mm-wide discs of fresh fungus-containing agar were excised from the culture margins and put in the center of the dishes, and then incubated at 28 °C in the dark for 7 days. For each colony, the colony mycelium was washed with sterile water and dried in an oven to a constant weight. Then, the dry weight was determined with an electronic balance. 

According to the method of Tan et al. [62], Bilay’s medium was amended without N as a control and with N as NH_4_^+^ or NO_3_^−^ at 1 mM or 5 mM, respectively. NH_4_^+^ was added in the form of (NH_4_)_2_SO_4_, and NO_3_^−^ was added as Ca(NO_3_)_2_. The conidial suspension of FOC was diluted to no more than 1000 conidia per milliliter with sterile distilled water, and the diluted suspension (0.1 mL) was inoculated into 500-mL Erlenmeyer flasks containing 250 mL of amended Bilay’s medium and incubated at 28 °C for 7 days with rotary shaking at 170 rev/min. Subsequently, the conidia were counted using a hemocytometer.

Czapek Dox medium was amended without N as a control and with N as NH_4_^+^ or NO_3_^−^ at 1 mM or 5 mM, respectively. NH_4_^+^ was added in the form of (NH_4_)_2_SO_4_, and NO_3_^−^ was added as Ca(NO_3_)_2._ Then, FOC was inoculated into the amended Czapek Dox medium (250 mL in 1-L flasks) and incubated at 28 °C on a rotary shaker (180 rpm) for approximately 10 days. The cultures were filtered through 0.45-mm membrane filters to exclude mycelium and micro-conidia. Subsequently, the filtrates were acidified with 2 M HCl to pH 2.5 and extracted three times with the same volume of ethyl acetate. The organic phase (ethyl acetate) was pooled and lyophilized under a vacuum. The residue was dissolved in 2 mL of methanol to obtain the crude toxin solution.

### 4.5. Pathogen Incubation and Infection

*The pathogen Fusarium oxysporum* f. sp. *cucumerinum* J. H. Qwen (FOC-NJAU-2) [63] was isolated from infected cucumber plants and provided by the Laboratory of Plant-Microbe Interactions of Nanjing Agricultural University in China. The FOC isolates were first incubated on potato dextrose agar medium (PDA) in Petri dishes at 28 °C in the dark for 7 days. Then, 8-mm-wide discs of fungus-containing agar were excised from the culture margins and inoculated into 1-L Erlenmeyer flasks containing Bilay’s medium [62]. The flasks were incubated at 28 °C for 7 days with rotary shaking at 170 rev/min. The resulting fungal cultures were filtered through four layers of sterile cheesecloth to eliminate mycelia fragments and centrifuged at 8000× *g* for 20 min to pellet the conidia. Then, the conidia were resuspended in sterile water and counted using a hemocytometer. For inoculation, the roots of four-week-old plants were immersed in FOC conidial suspension containing 10^7^ spores·mL^−1^ for 2 h. In the control, sterile distilled water was used in place of the conidial suspension. After inoculation, the plants were grown in hydroponic culture as described above.

### 4.6. Determination of the Disease Index

Plants were inspected for wilt symptoms daily for up to 2 weeks after inoculation. According to the method described by Wang et al. [64], the disease index of infected plants was rated on a scale of 0–4 (0: whole plant was healthy; 1: <25% of leaves wilted; 2: 25%–50% leaves wilted; 3: 50%–75% of leaves wilted; and 4: 75%–100% of leaves wilted or dead). The disease index was calculated according to the formula

$$\mathrm{Disease}\;\mathrm{index}\;(\mathrm{DI})\;=\frac{\sum \;(\mathrm{disease}\;\mathrm{grade}\;\times \;\mathrm{no}.\;\mathrm{of}\;\mathrm{plants}\;\mathrm{in}\;\mathrm{each}\;\mathrm{grade})}{\left(\mathrm{total}\;\mathrm{no}.\;\mathrm{of}\;\mathrm{plants})\;(\mathrm{highest}\;\mathrm{disease}\;\mathrm{grade}\right)}\times 100$$

### 4.7. FA Extraction and Analysis

FA was extracted according to the method described by Smith and Sousadias [57]. Tissues were weighed and homogenized with 50 mL of MeOH/1% KH_2_PO_4_ (1:1, *v*/*v*, pH = 3.0). Then, the suspension was centrifuged for 20 min at 20,000× *g*, and the pH of the supernatant was adjusted with 2 M HCL to 3.0. The acidified supernatant was sequentially extracted three times with methylene chloride. The methylene chloride extracts were pooled and evaporated to dryness at 40 °C on a rotary evaporator under vacuum. The residue was re-dissolved in 2 mL of methanol and analysed by high-performance liquid chromatography (HPLC).

FA was identified using a 1200 Series HPLC System (Agilent Technologies, Wilmington, DE, USA) equipped with an XDB-C18 column (4.6 mm × 250 mm, 5 μm, Agilent, USA) and set to 50 °C. The mobile phase for this analysis was (68:32) methanol-0.43% o-phosphoric acid at a flow rate of 1 mL/min. FA was detected by monitoring the UVA at 271 nm, the sample solutions (20 μL) were eluted for 15 min, and the retention time was approximately 5.8 min. The samples were quantified against a standard curve of synthetic FA (Sigma Chemical, St. Louis, MO, USA).

### 4.8. Specific Detection of FOC by Real-Time PCR

DNA from diseased cucumber plants was extracted by grinding 500 mg of three plant tissues (roots, stems, and leaves) in a mortar with liquid N according to the method described by Tjamos [65]. Then, the isolated DNA samples were used as templates for the polymerase chain reaction. *F. oxysporum* f. sp. *cucumerinum*-specific primers (FocF3 (F) 5′-AAACGAGCCCGCTATTTGAG-3′/FocR7 (R) 5′-ATTTCCTCCACATTGCCATG-3′) were used in a real-time PCR assay [66]. Real-time PCR amplification was performed with an ABI PRISM 7500 Sequence Detection System (Applied Biosystems, Vienna, Austria) in a total volume of 20 μL containing 10 μL of SYBR^®^ Premix Ex Taq™ (2×), 0.4 μL of ROX Reference Dye II (50×), 0.4 μL of each primer, 2 μL of DNA template, and 6.8 μL of sterile water. The PCR programme was 94 °C for 3 min, followed by 29 amplification cycles of denaturation at 94 °C for 45 s, 58 °C for 45 s and at 72 °C for 1 min. The specificity was examined by generating a dissociation curve after amplification. The melting curve was obtained at the end of the PCR run. The standard curves were generated according to a previous report [63], and the abundances of FOC were expressed as the copy concentration, as described previously by Cao et al. [63].

### 4.9. FA Response Experiment

Four-week-old cucumber plants cultured as described above were selected for the experiment, and the fungal toxin FA (Sigma) was diluted with the nutrient solution and supplied at a concentration of 60 ppm. Eight treatments were set up: 1AH, 5AH (mock-treated 1 mM or 5 mM NH_4_^+^-fed plants, respectively); 1NH, 5NH (mock-treated 1 mM or 5 mM NO_3_^−^-fed plants, respectively); 1AF, 5AF (60 ppm FA-treated 1 mM or 5 mM NH_4_^+^-fed plants, respectively); and 1NF, 5NF (60 ppm FA-treated 1 mM or 5 mM NO_3_^−^-fed plants, respectively). Cucumber plants were treated with FA at 60 ppm or mock treated for 9 h (7 h light and 2 h dark). The selected FA concentration was amended according to the report of Wang et al. [61]. The relative leaf membrane injury and FA content of the different position of the plants were measured after sampling. The methods of FA extraction and analysis are described above.

### 4.10. Relative Leaf Membrane Injury

Discs that were 10 mm in diameter without a midrib were cut from fully expanded leaves in each plant and washed with distilled water to remove any electrolytes that had adhered to leaves or were released from the cut tissue. The discs were infiltrated with distilled water under vacuum for 20 min. Then, the conductivity of the diffusate was directly measured using an electrical conductivity meter (DDS-11A, Leici Instrument Co., Shanghai, China). Subsequently, the leaf tissue was boiled in a 100 °C water bath for 20 min, and then a second conductivity measurement was performed after the sample had cooled to room temperature. Membrane injury was expressed as relative injury (%), which was defined as the ratio of the first conductivity measurement to the second.

### 4.11. Statistical Analysis

An analysis of variance was carried out using the SAS software package Version 6.12 (SAS Institute, Inc., Cary, NC, USA). Differences between treatments were determined by the *t* test, and *p* < 0.05 was used to indicate statistical significance.

## Figures and Tables

**Table 1:** Effects of different nitrogen sources on the dry weights of cucumber plants after FOC inoculation.

Treatments	Root (g)	Stem (g)	Leaf (g)
1A	0.54 ± 0.03 ^d^	1.21 ± 0.03 ^cd^	1.68 ± 0.08 ^c^
5A	0.73 ± 0.02 ^b^	1.96 ± 0.20 ^b^	2.58 ± 0.10 ^b^
1N	0.61 ± 0.01 ^c^	1.40 ± 0.14 ^c^	1.97 ± 0.21 ^c^
5N	0.85 ± 0.03 ^a^	2.66 ± 0.33 ^a^	3.81 ± 0.58 ^a^
1AI	0.48 ± 0.02 ^e^	1.07 ± 0.05 ^d^	1.18 ± 0.11 ^d^
5AI	0.36 ± 0.07 ^f^	1.38 ± 0.27 ^c^	1.62 ± 0.28 ^c^
1NI	0.57 ± 0.04 ^cd^	1.18 ± 0.11 ^cd^	1.63 ± 0.09 ^c^
5NI	0.80 ± 0.05 ^a^	2.39 ± 0.14 ^a^	3.64 ± 0.21 ^a^

Notes: 1A, 5A (non-inoculated control plants with 1 mM or 5 mM NH_4_^+^, respectively); 1N, 5N (non-inoculated control plants with 1 mM or 5 mM NO_3_^−^, respectively); 1AI, 5AI (inoculated plants with 1 mM or 5 mM NH_4_^+^, respectively); and 1NI, 5NI (inoculated plants with 1 mM or 5 mM NO_3_^−^, respectively). The data are the means ± SD of four replicates. Significant differences (LSD test, *p* < 0.05) of the same parts of the plants among treatments are indicated by different letters.

**Table 2:** Effects of different nitrogen sources on the FA distribution of cucumber plants after FA treatment.

Treatments	Root (μg·g^−1^ FW)	Stem (μg·g^−1^ FW)	Leaf (μg·g^−1^ FW)
1AF	195 ± 9 ^c^	273 ± 12 ^a^	142 ± 6 ^b^
5AF	206 ± 21 ^c^	238 ± 14 ^b^	174 ± 14 ^a^
1NF	270 ± 13 ^b^	205 ± 7 ^b^	120 ± 10 ^c^
5NF	306 ± 17 ^a^	168 ± 12 ^bc^	100 ±5 ^d^

Notes: plants were treated with 60 ppm FA and FA content was detected at 7 h after FA treatment when wilt symptoms occurred in 1 mM NO_3_^−^-fed plants. 1AF, 5AF (FA-treated 1 mM or 5 mM NH_4_^+^-fed plants, respectively); and 1NF, 5NF (FA-treated 1 mM or 5 mM NO_3_^−^-fed plants, respectively). The data are the means ± SD of four replicates. Significant differences (LSD test, *p* < 0.05) of the same parts of the plants among treatments are indicated by different letters.
